# Uncovering the barriers to exclusive breastfeeding for mothers living in Dhaka’s slums: a mixed method study

**DOI:** 10.1186/s13006-018-0186-5

**Published:** 2018-09-26

**Authors:** Halima Khatun, Carly A Comins, Rajesh Shah, M Munirul Islam, Nuzhat Choudhury, Tahmeed Ahmed

**Affiliations:** 1 Nutrition and Clinical Services Division, International Centre for Diarrhoeal Disease Research, Bangladesh (icddr,b), Mohakhali, Dhaka, 1212 Bangladesh; 2 James P. Grant School of Public Health, BRAC University, Mohakhali, Dhaka, 1212 Bangladesh; 3 Save the Children, Kathmandu, 44600 Nepal

**Keywords:** Initial breastfeeding, Exclusive breastfeeding, Associated factors, Barriers and facilitators, Mode of delivery, Slum, Dhaka, Bangladesh

## Abstract

**Background:**

Despite the substantial impact on child and maternal health, breastfeeding practices for infants remain at the suboptimum level in Bangladesh. Yet the understanding of why these practices are suboptimal, especially surrounding urban slum dwelling mothers, is unclear. The purpose of this study was to assess early infant feeding practices, examine associations with maternal factors, and uncover the facilitators and barriers to early feeding practices in selected slums of Dhaka, Bangladesh.

**Methods:**

A mixed method study was conducted from June to September 2016 using both quantitative and qualitative methods among mothers with children under the age of 6 months. The survey included 342 mother-infant pairs and 18 in-depth interviews were conducted. Univariate and multiple logistic regression was used to determine status of early infant feeding practices and factors associated with exclusive breastfeeding (EBF) within the previous 24 h. Transcripts were coded to uncover the facilitators and barriers surrounding early infant feeding practices.

**Results:**

Sixty four percent (220/342) of mothers initiated breastfeeding within 1 h, 96.5% (330/342) reported feeding colostrum, and 36.3% (124/342) infants were EBF in the last 24 h. After adjusting for child gender, maternal age, education, diet and household income; infant’s age (adjusted odds ratio (AOR) for 61–120 days 6.42; 95% CI 3.42, 12.1; AOR for 121–180 days 45.6; 95% CI 18.33, 113.45), prelacteal feeding (AOR 2.53; 95% CI 1.14, 4.58), lack of planning for EBF during pregnancy (AOR 4.06; 95% CI 1.09, 15.12) and infants delivered by cesarean section (AOR 2.76; 95% CI 1.34, 5.67) were negatively associated with EBF. During the 18 interviews, eight mothers reported a cesarean delivery and none of these mothers initiated breastfeeding within 1 h or exclusively breastfed. Moreover, all eight mothers gave their infants prelacteal feeds.

**Conclusions:**

The status of early infant feeding practices in Dhaka’s slums was poor. The negative impact of cesarean section on all early infant feeding practices was evident in both quantitative and qualitative analysis.

## Background

Appropriate feeding for first 6 months of life is crucial for child health and survival. Breastfeeding saves lives and promotes physical and mental health throughout childhood and beyond [1–5]. Increasing infant survival by at least six times, breastfeeding impacts childhood survival significantly [1]. Moreover, infants exclusively breastfed are 14 times less likely to die in first 6 months compared to non-breastfed infants, while partially and non-breastfed children are at 5–9 times higher risk of death due to infection [1]. If breastfeeding was scaled up to near universal level (90–95%) 823,000 deaths could have been saved in low and middle-income countries (LMICs) in 2015 [6].

Despite the known positive impact of breastfeeding on infant survival and health, the rate of exclusive breastfeeding globally is low [7]. In 2015, 37% infants less than 6 months old were exclusively breastfed in LMICs [6]. In Bangladesh, for the past two decades, the prevalence of exclusive breastfeeding remained constant around 55% [8]. Additionally, in 2014 the rate of early initiation of breastfeeding was 51% and 27% of the newborn received prelacteal feeds in their first 3 days of life [8].

Bangladesh is undergoing a rapid urbanization process. The current urban population will increase by 50% in 2028. One third of this urban population live in slums and are inclined to experience negative factors of health and nutrition. According to Bangladesh Urban Health Survey 2013, health indicators are lower in urban slums compared to non-slum areas [9]. Among infants of mothers from Dhaka’s slum, 10% of infants were exclusively breastfed for 6 months, breastfeeding was initiated within 1 h in 24% of infants, and 54% of infants were given prelacteal feeds; which are much lower compared to the national rates of 55% EBF, 51% early initiation and 27% prelacteal feeds [8, 10].

Low rates of early initiation and exclusive breastfeeding are reflective of various factors influencing a mothers’ inability or reluctance to breastfeed. A mother’s experience, and in turn her child bearing and rearing, is largely embedded in social, cultural, and personal norms and perceptions. Therefore, it is difficult to generalize the determinants of early infant feeding practices. Exclusive breastfeeding for first 6 months has been found to be associated with maternal age, education, occupation, economic status, place of residence, and mother’s intention to breastfeed [11–20]. Other factors influencing exclusive breastfeeding are history of prelacteal feeding, early initiation, antenatal care visits, prenatal counseling, influence from healthcare providers, type of delivery [11, 13, 14, 16, 19, 20].

Specifically, a birth by cesarean section negatively impacts early infant feeding practies, and globally the rate of cesarean delivery is increasing. The largest absolute increase in cesarean delivery has been observed in developing countries, with an increase of 6.7% from 1990 to 2014 [21]. Moreover, within Bangladesh, cesarean delivery has increased from 4% in 2004 to 23% in 2014 [8]. The implications of cesarean delivery on early infant feeding is yet to be explored within the Bangladesh context.

Moreover, qualitative studies found that maternal perception influenced early infant feeding practices in different areas of Bangladesh and other low and middle income countries [22–25]. Such influencing factors vary, not only in the context of different countries but also in different settings within country. An explanatory study conducted among slum dwellers in Dhaka revealed poor practice of initiation of breastfeeding within 1 h of birth [25]. Still the perceived barriers and facilitators of exclusive breastfeeding among the mothers with children less than 6 months of age need to be explored. A recent study from Bangladesh Urban Health Survey (BDHS) 2014 data revealed that less educated and housewife mothers are more inclined to exclusive breastfeeding [26]. Therefore, given the limited evidence surrounding feeding practices in Dhaka’s slums, the study investigated the status of early feeding practices in infants less than 6 months, examined factors associated with such practices, and uncovered the facilitators and barriers to early infant feeding practices in selected slums of Dhaka, Bangladesh.

## Methods

### Study design and sample selection

A mixed method study was conducted using both quantitative and qualitative methods in two conveniently selected slums, Korail and Sat Tala slum, in Dhaka. Korail slum is one of the largest slums and has approximately 15,000 households, while Sat Tala slum has nearly 6500 households, and 342 mothers with children under the age of 6 months were studied. Household listing in the selected area was carried out to identify the household with eligible participants prior to interview. All eligible households with children less than 6 months in the selected area were visited and mother infant dyads were interviewed and evaluated for nutritional status. Qualitatively, 18 mothers were purposively selected from both slums and interviewed with an in-depth interview guide.

Sample size calculation: Sample size for quantitative method was calculated based on current exclusive breastfeeding rate as an indicator of early infant feeding practice at 90% confidence level, 5% precision and with a design effect of 1.2 [8]. Estimated sample size was 324 and with a 10% attrition rate required sample size was 360.

### Selection of variables

Independent variables were selected based on extensive literature review. Relevant child characteristics for early infant feeding were child age, gender, birth order, immunization status and infant’s nutritional status. Maternal characteristics assessed were age, education, occupation, age at first pregnancy, planning for EBF during pregnancy, maternal depressive symptoms, number of antenatal care visit, postnatal care visit, place of delivery, assisted delivery by trained professionals, mode of delivery, maternal dietary diversity (24-h recall), maternal nutritional status, initiation of breastfeeding and practice of giving prelacteal feeds. Household characteristics considered were monthly income, asset index as a proxy indicator of household wealth, and household food consumption (7 days recall). Exclusive breastfeeding coded as a binary outcome and used as a dependent variable.

### Data collection

Face-to-face structured questionnaires were administered to mothers on infant feeding practices at birth and within the last 24 h. Early infant feeding practices were summarized as exclusively breastfed (consumed only breast milk with an exception of Oral Rehydration Solution (ORS), medicine drops, syrups); predominantly breastfed (consumed breast milk with certain liquids (water and water-based drinks, fruit juice, ritual fluids and ORS, drops or syrups (vitamins, minerals, and medicines); partially breastfed (consumed breast milk with any food or liquid); and non-breastfed (did not consume breast milk) [27]. Other information collected included sociodemographic characteristics of infant, mother and household; maternal psychological condition; environmental factors including family, workplace and social service facilities; maternal and household diet; and anthropometric measurement of infants and mothers. For qualitative data collection individual in-depth interview was conducted. A semi-structured questionnaire adopted from ProPAN 2 was used to guide the interview ProPAN is a set of research tools designed for ministries of health (MoHs), nongovernmental organizations (NGOs), and bilateral and international organizations working to improve the diets and feeding practices of children under 24 months old to prevent early childhood malnutrition [28]. ProPAN multi-module field manual provides detailed instructions on how to collect, analyze, and integrate the quantitative and qualitative data required to design and evaluate interventions. The study period was from June to September 2016.

Derived variable for analysis: Maternal depressive symptoms were measured by a 20 items self-reporting questionnaire developed by World Health Organization and previously used nationally in Bangladesh [29]. Mothers with a score of eight positive answers were categorized as having depressive symptoms. Dietary diversity was based on a 24-h recall of food consumption. Food was captured within nine pre-determined food groups, as defined by Food and Nutrition Technical Assistance (FANTA). Mothers consuming five or more food groups were categorized as having adequate dietary diversity in terms of macro- and micro-nutrients.

Household food consumption, a proxy indicator of household food security developed by World Food Program, has been validated in Bangladesh and was used in the 2009 Bangladesh Household Food Security and Nutrition Assessment Report. This composite score was based on a seven-day recall of frequency of food consumption and categorized as poor consumption (0–28), borderline consumption (28.5- < 42), acceptable low consumption (42–52) and acceptable high consumption (> 52) [30].

Household asset index was calculated using household asset data on ownership of several consumer items, access to safe drinking water, and improved sanitation [8], and households were indexed into quintiles from poorest to richest.

### Data analysis

#### Quantitative

Data was analyzed with Stata SE Version 13.0 software. Descriptive analysis was conducted to characterize the study population by infant, maternal and household characteristics. Simple logistic regression was utilized to determine the association between each independent variable and the outcome variable, exclusive breastfeeding status, and for consideration in the final model. Results are shown as crude odds ratios and *p*-values. Variables considered for inclusion in the multiple logistic regression analysis were selected based on previous literature [12–15, 17–20]. Variance inflation factor (VIF) was calculated to detect multicollinearity among the variables. If the VIF of a factor was 5–10 it was considered as moderate collinearity. If the VIF > 10 it was considered severe collinearity and the predictor was removed from the model. The model with highest R square value was considered to be the final regression model. All variables with a *p*-value less than 0.15 in bi-variate analysis were included, after adjusting for other variables [31]. Age and gender were considered as non-modifiable factors and adjusted in the final model [31]. Associated factors with *p*-value < 0.05 was considered to be statistically significant.

#### Qualitative

To explore the facilitators and barriers to optimal early infant feeding practices for the first 6 months of life, 18 in-depth interviews with mothers were conducted in the selected slums. The sample included purposively selected participants who completed the quantitative questionnaire. Nine mothers were selected who exclusively breast fed and nine who did not, based on reported exclusive breastfeeding status within 24 h. Two trained interviewers interviewed the participants per an in-depth interview guideline that included questions surrounding ideal early infant feeding practices during the first 6 months of life [28].

All interviews were tape recorded and transcribed in Bangali the same day. Qualitative analysis was conducted thematically. Transcripts were manually reviewed and coded for topics related to infant feeding practices. Transcripts were double coded and agreement of any discordant code was achieved through discussion. Finally, a matrix was prepared based on the coding and translated into English. Barriers and facilitators were categorized in external and internal factors in the matrix. External factors were those over which the caregiver has little (if any) control and internal factors refers to those intrinsic to the caregiver [28]. No software was used for the analysis.

## Results

### Descriptive characteristics of the participants (from quantitative survey)

**Fig. 1:**
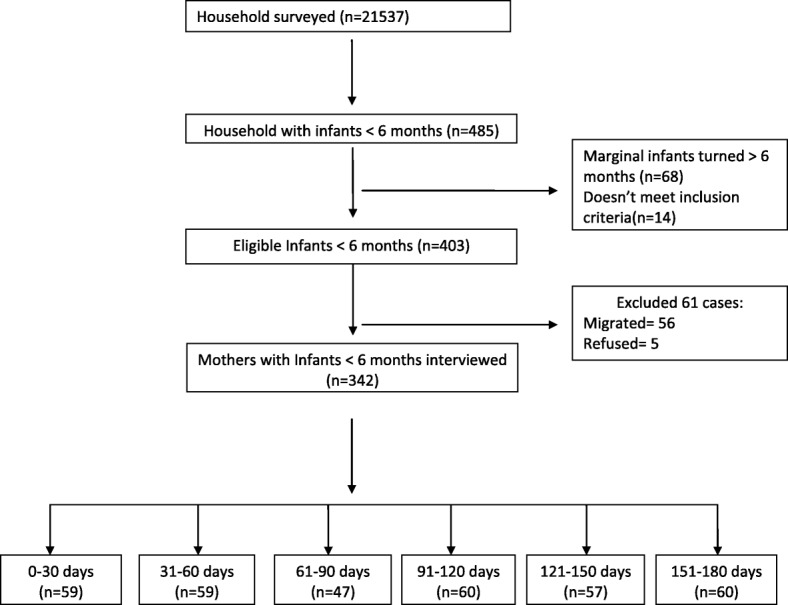
Study profile

A total of 342 mothers with infants less than 6 months of age were interviewed in this study, and a study profile is described in Fig. 1. A descriptive analysis of the sample, stratified by early infant feeding practices, is presented in Table 1. The majority of mothers (90.6%) reported being housewives, and 42.9% had received primary level education. Based on 24 h recall, 52.1% of mothers’ diet lacked adequate dietary diversity (consumption of ≥5 groups). Moreover, while 70% of mothers had normal nutritional status, 10.5% were thin and 22.5% overweight/obese (BMI <  18 and BMI >  25, respectively). Among the infants, 13.7% were stunted, 7.6% were wasted, 10.8% were underweight, and 4.7% were overweight.

**Table 1:** Descriptive statistics of the sample stratified by percentage of exclusively breastfed, predominantly breastfed, partially breastfed and non-breastfed

	*n*	Exclusively breastfed % (95% CI)	Predominantly breastfed % (95% CI)	Partially breastfed % (95% CI)	Non breastfed % (95% CI)
Infants characteristics					
Infant’s age					
0–60 days	118	70.3(61.4,78)	13.6(8.4,21.1)	16.1(10.4,24)	0(0,0)
61–120 days	107	29.9(21.9,39.4)	24.3(17,33.5)	42.1(32.9,51.7)	3.7(1.4,9.7)
121–180 days	117	7.7(4,14.2)	28.2(20.7,37.2)	61.5(52.3,70)	2.6(0.8,7.8)
Gender					
Male	178	38.2(31.3,45.6)	23(17.4,29.9)	36(29.2,43.3)	2.8(1.2,6.6)
Female	164	34.1(27.2,41.8)	20.7(15.2,27.7)	43.9(36.4,51.7)	1.2(0.3,4.8)
Birth order (Reference child)					
1st child	145	37.2(29.7,45.5)	26.2(19.6,34.1)	34.5(27.1,42.7)	2.1(0.7,6.3)
2nd child or over	197	35.5(29.1,42.5)	18.8(13.9,24.9)	43.7(36.8,50.7)	2(0.8,5.3)
Immunization status (According to mothers’ response)					
Properly Immunized	263	29.3(24.1,35.1)	26.2(21.2,31.9)	42.2(36.3,48.3)	2.3(1,5)
Not immunized	79	59.5(48.1,69.9)	7.6(3.4,16.1)	31.6(22.2,42.9)	1.3(0.2,8.8)
Maternal characteristics					
Age					
< 18 years	21	38.1(19.1,61.7)	14.3(4.2,38.7)	47.6(26.3,69.8)	0(0,0)
≥ 18 years	321	36.1(31,41.6)	22.4(18.2,27.3)	39.3(34,44.7)	2.2(1,4.5)
Maternal education level					
No formal education	112	36.6(28.1,46)	20.5(14,29.2)	40.2(31.4,49.6)	2.7(0.9,8.1)
Primary complete	147	33.3(26.1,41.4)	21.8(15.8,29.3)	42.9(35,51.1)	2(0.7,6.2)
secondary or above	83	41(30.7,52)	24.1(16,34.7)	33.7(24.2,44.8)	1.2(0.2,8.4)
Maternal occupation					
Housewife	310	37.7(32.5,43.3)	22.9(18.5,27.9)	37.7(32.5,43.3)	1.6(0.7,3.8)
Working mother	32	21.9(10.3,40.4)	12.5(4.50,30.2)	59.4(40.9,75.5)	6.3(1.4,23.2)
Maternal age at 1st pregnancy					
< 18 years	98	34.7(25.8,44.8)	18.4(11.8,27.5)	45.9(36.2,56)	1(0.1,7.1)
≥ 18 years	244	36.9(31,43.2)	23.4(18.4,29.1)	37.3(31.4,43.6)	2.5(1.1,5.4)
EBF planning during pregnancy					
planned	308	38.3(33,43.9)	20.5(16.3,25.4)	39.6(34.3,45.2)	1.6(0.7,3.9)
not planned	34	17.6(7.8,35.2)	35.3(20.6,53.4)	41.2(25.4,59)	5.9(1.4,22)
Maternal depressive symptoms					
Absent	263	39.2(28.9,50.6)	17.7(10.7,28.0)	43(32.4,54.4)	0(0,0)
Present	79	35.4(29.8,41.4)	23.2(18.5,28.7)	38.8(33,44.8)	2.7(1.3,5.5)
Antenatal care (ANC)					
No or < 4 ANC visits	106	28.3(20.4,37.8)	24.5(17.2,33.8)	44.3(35,54)	2.8(0.9,8.6)
4 visits or more	236	39.8(33.7,46.3)	20.8(16,26.5)	37.7(31.7,44.1)	1.7(0.6,4.5)
Received postnatal care services	176	35.8(29,43.2)	21.6(16.1,28.4)	40.3(33.3,47.8)	2.3(0.8,6)
Place of delivery					
Home delivery	148	35.1(27.8,43.2)	25.0(18.6,32.7)	38.5(30.9,46.7)	1.4(0.3,5.3)
Facility delivery	194	37.1(30.6,44.2)	19.6(14.6,25.8)	40.7(34,47.8)	2.6(1.1,6.1)
Delivery assisted by					
Trained professionals	246	37.4(31.5,43.7)	21.1(16.5,26.7)	39.4(33.5,45.7)	2(0.8,4.8)
Traditional birth attendant	96	33.3(24.5,43.5)	24(16.4,33.7)	40.6(31.1,50.9)	2.1(0.5,8.1)
Mode of delivery					
Normal Vaginal Delivery (NVD)	256	37.5(31.7,43.6)	21.5(16.9,27)	39.1(33.2,45.2)	2(0.8,4.6)
Cesarean Section	86	32.6(23.4,43.3)	23.3(15.4,33.5)	41.9(31.7,52.7)	2.3(0.6,9.1)
Maternal dietary diversity					
≥ 5 food groups	164	41.5(34.1,49.2)	20.7(15.2,27.7)	36(28.9,43.7)	1.8(0.6,5.6)
< 5 group	178	31.5(25,38.7)	23(17.4,29.9)	43.3(36.1,50.7)	2.2(0.8,5.9)
Maternal BMI					
Normal (18.5–24.9)	229	39.3(33.1,45.8)	22.3(17.3,28.2)	37.6(31.5,44.1)	0.9(0.2,3.5)
Thin (< 18.5)	36	25(13.1,42.4)	11.1(4,27.1)	61.1(43.7,76.1)	2.8(0.4,18.7)
Overweight or obese (> 25)	77	32.5(22.8,43.9)	26(17.2,37.1)	36.4(26.2,47.9)	5.2(1.9,13.3)
Early initiation of breastfeeding					
≤ 1 h after birth	204	37.3(30.8,44.2)	20.6(15.5,26.7)	41.2(34.6,48.1)	1(0.2,3.9)
> 1 h after birth	138	34.8(27.2,43.2)	23.9(17.5,31.8)	37.7(29.9,46.1)	3.6(1.5,8.5)
Prelacteal history					
No prelacteal given	156	43.6(35.9,51.6)	19.2(13.7,26.3)	35.3(28.1,43.2)	1.9(0.6,5.9)
Gave prelacteal	186	30.1(23.9,37.1)	24.2(18.5,30.9)	43.5(36.5,50.8)	2.2(0.8,5.6)
Household characteristics					
Household income					
< 10,000	81	27.2(18.4,38.1)	25.9(17.4,36.8)	43.2(32.7,54.4)	3.7(1.2,11.1)
10,000 to < 20,000	186	38.2(31.4,45.4)	23.1(17.6,29.8)	37.1(30.4,44.3)	1.6(0.5,4.9)
> 20,000	75	41.3(30.6,53)	14.7(8.2,24.9)	42.7(31.8,54.3)	1.3(0.2,9.2)
Household head					
Female head	42	31(18.5,47)	16.7(7.9,31.8)	47.6(32.6,63.1)	4.8(1.1,18)
Male head	300	37(31.7,42.6)	22.7(18.3,27.8)	38.7(33.3,44.3)	1.7(0.7,4)
Household food consumption score					
Acceptable high consumption (> 52)	274	35.4(29.9,41.3)	21.9(17.4,27.2)	40.5(34.8,46.5)	2.2(1,4.8)
Acceptable food consumption (42–52)	44	47.7(33,62.8)	18.2(9.1,33)	34.1(21.3,49.7)	0(0,0)
Poor or borderline food consumption (≤ 42)	24	25(11,47.4)	29.2(13.7,51.5)	41.7(22.9,63.1)	4.2(0.5,27.4)
Toilet facility					
Improved facility	198	34.8(28.5,41.8)	22.2(16.9,28.6)	41.9(35.2,49)	1(0.2,4)
Non improved facility	144	38.2(30.5,46.5)	21.5(15.5,29.1)	36.8(29.2,45.1)	3.5(1.4,8.2)
OVERALL	342	36.3(31.3,41.5)	21.9(17.8,26.7)	39.8(34.7,45.1)	2.1(1,4.3)

*CI* Confidence Interval

Prior to giving birth, 90% of mothers planned to exclusively breastfeed for 6 months. Approximately, 66% of mothers received ≥4 visits antenatal checks and 51.5% received a postnatal care visit. Of the 342 mothers, 71.9% delivered by a trained professional at home or at a facility and 56.7% delivered at a healthcare facility. A quarter (25.1%) of mothers had a caesarian delivery.

At the household level, 54.4% of households had a monthly income of 10,000–20,000 BDT, and 87.7% reported a male household head. Based on a seven-day recall, 21.9% of households were found to have borderline to poor food consumption.

### Early initiation of breastfeeding

The study found 64.2% of mothers initiated breastfeeding within 1 h of birth. Seven of the 18 mothers who qualitatively reported early initiation explained that support from healthcare workers (i.e. midwives, traditional birth attendants, hospital staff) and in-laws (6 out of 7, 5 out of 7, respectively) facilitated their ability to initiate breastfeeding within 1 h. One mother, age 26 from Korail slum, was influenced “from TV, health worker and neighbors. .. mother and midwives told me to feed the baby and I do so. .. If midwives and family member help to hold the baby it becomes easier for mother to feed the baby early” [101062]. Two out of seven mothers mentioned about learning of early initiation of breastfeeding from TV programming.

Qualitative analysis found that 11 mothers out of 18 did not initiate breastfeeding within 1 h. The main reason for failure of early initiation was because the mother had a cesarean delivery and were unconscious, experiencing side effects of surgery, or unable to produce milk (8 out of 11 mothers). One mother, aged 23 from Korail slum, explained, “after cesarean section I was sick and there was no milk secretion for three days. .. when they gave the baby to me after three to four hours I put the baby on the breast but there was no milk” [101350]. Of the other three mothers who did not early initiate, one mother had incorrect knowledge surrounding early initiation and two, although delivered normally, could not initiate due to the lengthy infant cleaning time at the facility.

The importance of giving colostrum was widely mentioned by the mothers and 96.5% reported feeding colostrum to their infants. Qualitatively, a total of nine out of the 18 mothers stated they administered colostrum during the first 3 days. But only five of these nine mothers provided solely colostrum to their infant during the first 3 days of life, the rest also provided prelacteal feeds.

Moreover, 54.4% of mothers reported prelacteal administration, providing anything other than breastmilk to child during the first 3 days of life. Honey (23.4%), sugar water (13.7%), plain water (10.8%), and infant formula (9.9%) were the most common prelacteal feeds provided to infants. Of the 18 mothers who qualitatively responded, 11 mothers reported administering prelacteal feeds. Moreover, of these 11 mothers, five did so because of the perception of insufficient milk production and to stop the infant from crying. Formula feeding during the first 3 days after birth was mainly due to post cesarean section outcomes (sickness, unconsciousness, and/or no milk production yet) and per the doctors’ prescription. Six of the eight mothers who had a cesarean delivery, administered formula because there was no early initiation of breastfeeding and milk production has not started yet. A mother 23 years old from Korail slum described, “[the] doctor said I can breastfeed my baby but I was sick and irritated. Could not move properly. Even though I tried to breastfeed but baby was crying, I felt frustrated. They prescribed formula and I brought the formula from the hospital and fed him” [101350]. Reasons for prelacteal administration of honey or sugar water was to clear the voice, prevent cold, and sweeten the speech of the child in future. Plain water was given because mothers perceived that infant was thirsty and water would keep the child hydrated. Moreover, seven of the 18 mothers qualitatively said they gave mustard oil to infant to clear mouth and throat after birth.

### Exclusive breastfeeding (EBF)

**Fig. 2:**
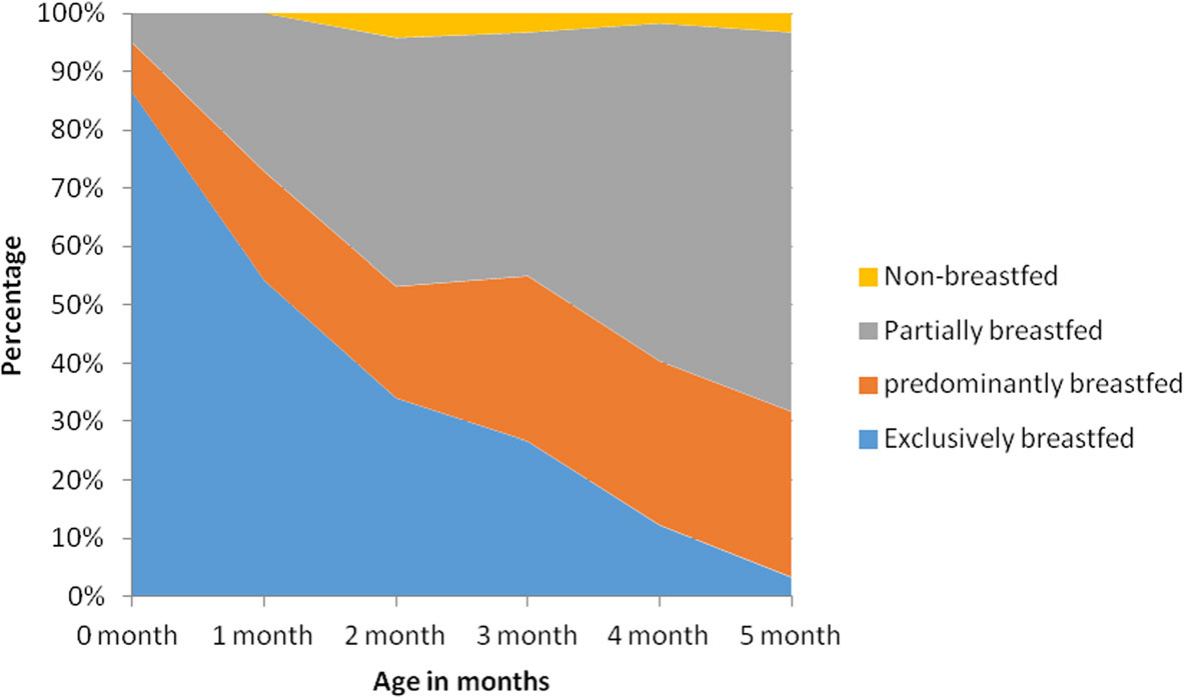
Infant feeding practices by age (Area Plot)

Overall 36.3% infants were exclusively breastfed within the prior 24 h and the prevalence of exclusive breastfeeding was found to decrease with the infant’s age (Fig. 2). Infants 2–4 months and 4–6 months had 5.6 (95% CI 3.14, 9.85) and 28.5 (95% CI 12.96, 62.48) times higher odds of not being exclusively breastfed, respectively, compared to infants 0–2 months. The proportion of infants predominantly, partially, and non-breastfed was 21.9%, 39.8%, and 2.1% respectively (Table 1). Common substitutes or supplements provided include plain water (52.9%), infant formula (27.2%) and homemade thin porridge (9.7%).

We found that mothers who had no or less than four antenatal care visits (OR 1.68; 95% CI 1.02, 2.76), not intending to exclusively breastfeed when pregnant (OR 2.9; 95% CI 1.17, 7.21), administered prelacteal feeds (OR 1.79; 95% CI 1.15, 2.8) and had low dietary diversity (OR 1.54; 95% CI 0.99, 2.4) were less likely to be exclusively breastfed. Infants from households with a higher asset score (OR 0.73; 95% CI 0.58, 0.9) were less likely to be non-exclusively breastfed (Table 2). There was no association found between maternal nutritional status and exclusive breastfeeding.

**Table 2:** Factors associated with non-exclusive breastfeeding of infants less than 6 months

Independent variables	OR (95% CI)	*p* - value	Adjusted odds Ratio (95% CI)	*p* - value
Age of infants				
0–60 days	Reference			
61–120 days	5.56 (3.14,9.85)	0.00	6.42 (3.42,12.07)	0.00
121–180 days	28.46 (12.96,62.48)	0.00	45.6 (18.33,113.45)	0.00
Child gender				
Female	Reference			
Male	0.84 (0.54,1.31)	0.44	0.81 (0.46,1.45)	0.48
Maternal age				
≥ 18 years	Reference			
< 18 years	0.92 (0.37,2.28)	0.86	0.39 (0.12,1.22)	0.10
Maternal education				
No formal education	Reference			
Primary complete	1.15 (0.69,1.93)	0.58	1.82 (0.91,3.63)	0.09
Secondary or higher	0.83 (0.46,1.49)	0.54	2 (0.89,4.51)	0.10
Antenatal visits				
4 or more visits	Reference			
No or < 4 visits	1.68(1.02,2.76)	0.04	1.41 (0.72,2.76)	0.31
Planning for EBF				
Planned EBF during pregnancy	Reference			
Did not plan EBF	2.9(1.17,7.21)	0.02	4.06 (1.09,15.12	0.04
Mode of delivery				
Normal vaginal delivery	Reference			
CS	1.24(0.74,2.08)	0.41	2.76 (1.34,5.69)	0.01
Prelacteal history				
No prelacteal	Reference			
Prelacteal given	1.79(1.15,2.8)	0.01	2.53 (1.4,4.58)	0.00
Maternal Dietary Diversity (MDD)				
Acceptable dietary diversity (> = 5 groups)	Reference			
Low DD (< 5 food groups)	1.54(0.99,2.4)	0.06	1.55 (0.87,2.78)	0.14
Asset score	0.73(0.58,0.9)	0.00	0.81 (0.59,1.1)	0.18
HH income				
< 10,000 BDT				
10,000–20,000 BDT	0.6(0.34,1.07)	0.08	0.48 (0.22,1.03)	0.06
> 20,000 BDT	0.53(0.27,1.04)	0.06	0.38 (0.14,1.04)	0.06

*OR* Odds Ratio, *CI* Confidence Interval

In the final regression model, after adjusting for maternal age, child age and gender and household asset score, infant’s age (AOR for 2–4 months: 6.42; 95% CI: 3.42, 12.1; AOR for 4–6 months: 45.6; 95% CI 18.33, 113.45), prelacteal history (AOR 2.53; 95% CI 1.14, 4.58), not intending to exclusively breastfeed when pregnant (AOR 4.06; 95% CI 1.09, 15.12), and a cesarean delivery (AOR 2.76; 95% CI 1.34, 5.67) were all found to be risk factors for an infant not being exclusively breastfed (Table 2).

Mothers were asked if they fed their infant anything other than breastmilk since birth. Six of the 18 mothers reported exclusively breastfeeding their child. The main facilitators mentioned by the mothers were correct knowledge regarding the importance of exclusive breastfeeding, support from family, and advice and recommendation to breastfeed from health personnel (Table 3). One mother, age 19 from Korail slum, said, “. .. my parents, in-laws and neighbors also suggested me not to give anything else before six months. Sometimes I think only breastmilk is not sufficient for my baby and feel like she needs other milk or food. But I do not give other milk because doctor said not to do so” [101150]. Other facilitators mentioned by the mothers were sufficient breast milk and correct knowledge regarding the importance of exclusive breastfeeding (Table 4).

**Table 3:** Barriers and facilitators for early initiation; feeding colostrum and not giving any prelacteal feeds

Barriers		Facilitators	
Internal	External	Internal	External
Thinks that breast milk become available only after 2–3 days Mother didn’t know that breast milk should be given within 1 h Put baby to breast but there was no milk secretion	Mother was sick/unconscious took too long to bring the baby to mother after surgery Mother had difficulties to hold the baby to feed after surgery It took too long to clean the baby child was very sleepy child didn’t demand	If mother is well Mother knew about early initiation Previous experience Knew colostrum should be given Mother trained from NGO	Normal delivery Midwives assist to put baby on the breast Grandmother/other family member/ doctor/ midwives suggest to give breast milk as early as possible Heard this from health workers/TV/ text book
Thinks that honey (clear voice/prevent cold), mustard oil (to clear mouth and throat), sugar water (to clear cough) is required Did not know that other substance Should not be given no breastmilk before 3 days after delivery	Midwives suggested honey Grandmother gave what she feels better for the child Mother was sick/unconscious Doctor /nurse/midwives prescribed infant formula Grandmother gave sugar water as believed breast milk was insufficient Child was crying too much	Thinks only colostrum should be given up to 3 days after birth Knew that baby should not be given anything except breast milk	Doctor/ nurse/ midwives suggest not to give anything but breast milk Another wet nurse mother fed baby when mother was sick

**Table 4:** Perceived barriers and facilitators for exclusive breastfeeding

Barriers		Facilitators	
Internal	External	Internal	External
Insufficient breast milk (thinks due to inadequate diet of mother, medicine after surgery, infrequent feeding) Mother left for work Thinks baby should drink water as it feels thirsty Thinks complementary food should start after 4 months	Grandmother did not allow breastfeeding Doctor prescribed formula Bottle feeding Maternity leave < 6 months No crèche at the workplace Workload does not permit to breastfeed at workplace	Mother knew that child should be given only breastmilk up to 6 months Knew that other food is harmful for child health Sufficient breastmilk -Thinks breastmilk is best for baby	Doctor suggest that baby should not be given anything else before 6 months Breast milk is free of cost Grandmother/family member helps in household work Husband support breastfeeding -bottle feeding is hazardous in terms of preparation

Moreover, 12 of the 18 mothers interviewed reported failing to exclusively breastfeed for the first 6 months of the infant’s life. The majority of the mothers reported the perception of insufficient breastmilk as the main barrier towards exclusive breastfeeding, and mothers voiced that the infant’s cry was an indication of insufficient breastmilk. Reasons behind breastmilk insufficiency perceived by the mothers was inadequate maternal consumption of food, medicine taken after surgery, and infrequent breastfeeding. All eight mothers who had a cesarean section did not exclusively breastfeed. One mother, age 22 from Sattala slum explained, “I cannot give my baby only breast milk because after cesarean section I took antibiotics and my breast milk dried up. Baby does not get sufficient breast milk and cries a lot. Then I started Lactogen [formula]. .. baby does not want to suck the breast when there is insufficient milk” [102115]. Of the 12 mothers who did not exclusively breastfeed, nine provided their infant with formula. Seven of the 12 mothers fed their infant water, and these mothers noted the importance of exclusive breastfeeding but believed their infant was dehydrated and therefore needed water. An additional two mothers reported not being able to exclusively breastfeed due to their employment status.

## Discussion

This mixed method study investigated not only the status of infant feeding practices and the associated factors, but it also uncovered the main facilitators and barriers behind such practices among mothers with infants under 6 months in the slums of Dhaka, Bangladesh. The study found that one fourth of mother had a cesarean section, and this type of delivery impacted early initiation of breastfeeding, colostrum administration, and exclusive breastfeeding. Moreover, mothers who delivered by cesarean section qualitatively explained their inability to abide by early infant feeding best practices due to their unconsciousness, sickness, pain, and prescription medication post-surgery and the subsequent need to supplement breastfeeding due to delayed or inadequate milk production.

Early initiation of breastfeeding was found to be slightly higher in our study than compared to overall national average from BDHS 2014, 64.2% and 50.8%, respectively [8]. Qualitative study found only seven out of 18 mothers initiate breastfeeding within 1 h. Of the 11 mothers who did not, eight qualitatively said they failed to because of the cesarean section. This finding echoes the 2016 finding that only 37.7% of mothers who delivered in a health facility in Bangladesh initiated within 1 h [8], which may be reflective of type of delivery. Analysis of national survey data in Nepal showed that cesarean delivery is one of the risk factors for delayed initiation of breastfeeding [32]. Delayed breastfeeding initiation has also been experienced by women who had a cesarean section in other research as well [33–36]. Maternal inability to breastfeed their child just after surgery delayed the early initiation and promoted introduction of prelacteal feeds. Moreover, breastfeeding initiation within 1 h after delivery has also been found to be a predictor of breastfeeding continuation [37–39].

As seen in other studies, 96.5% of the infants in this study were fed colostrum, yet in the qualitative analysis only nine out of 18 mothers did so. Colostrum feeding was supplemented by prelacteal feeding practices, as has been found in previous studies [22, 40]. Honey and sugar water were most widely reported prelacteal feeds given to the infants in this study. In South East Asia this a common practice to use these foods as a first thing to be provided to infants after birth [22, 40, 41]. These are also widely used as prelacteal feeds in rural Bangladesh [22, 40]. We found that infants who received prelacteal feeds were 2.5 times higher risk of not being exclusively breastfed. Prelacteal administration was also reported as a risk factor for failure to exclusively breastfed in previous studies both in Bangladesh and in other developing countries [14, 42].

Prevalence of exclusive breastfeeding in the Dhaka’s slums was found to be lower (36%) than the national prevalence (55%) of exclusive breastfeeding [8]. However, this finding is consistent with the findings from other studies in rural and slum area of Bangladesh [13, 43]. As this study found, another cross-sectional study in rural Bangladesh found a negative association between cesarean delivery and exclusive breastfeeding [13]. In-depth interviews revealed that most of the mothers (12 out of 18) reported insufficient breast milk production as one of the main reasons for being unable to exclusively breastfeed. This was the case mentioned in other formative research done in Bangladesh [40]. Moreover, all of the eight mothers within the qualitative portion of the study who had a cesarean delivery did not exclusively breastfeed their child. Inability to buy/eat nutritious food and intake of medicine after surgery was mentioned as additional reasons for insufficient breast milk which is a misconception of mothers. Only one mother correctly mentioned that after surgery infrequent feeding of her child lead to insufficient breast milk which she realized later but by that time her child became habituated to bottle feeding. This maternal perception was also reported from rural Bangladesh and from different age groups [22]. With the increasing rate of cesarean delivery nationally, it is an alarming risk for optimal early infant feeding practices. Proper implementation of Baby Friendly Hospital Initiative could be one the solutions to combat this challenge [11, 17]. Additionally, post-cesarean pain management and assisted breastfeeding initiation is believed to be helpful in improving EBF rates.

A few limitations within this study need to be noted. The definition of EBF used here according to 24 h-recall period is subject to bias and misreporting. Additionally, the data collection was conducted during the heat of summer. Therefore, maternal perception of her infant’s thirst and subsequent administration of water may have been higher than normal.

## Conclusions

Early infant feeding practices among urban slum dwelling mothers in Dhaka, Bangladesh was found to be lower than the universal levels recommended by the WHO. Moreover, this study found that the cesarean section rate (25.1%) among these mothers was higher than the optimal rate for caesarean sections (10–15%) as put forth by the WHO. Cesarean delivery was found to be associated with failure to exclusively breastfeed and qualitative findings highlighted the difficulty surrounding cesarean delivery and adhering to recommended early infant feeding practices due to this type of delivery. This finding should be studied in greater depth to understand the national impact of increasing caesarean section delivery rates and the impact on early infant feeding practices as well as maternal and infant immediate and long term health.

## Abbreviations

ANC: Antenatal care
AOR: Adjusted Odds Ratio
BMI: Body Mass Index
CS: Cesarean Section
EBF: Exclusive breastfeeding
FANTA: Food and Nutrition Technical Assistance
icddr,b: International Centre for Diarrhoeal Disease Research, Bangladesh
LMICs: low and middle income countries
MDD: Maternal Dietary Diversity
NCSD: Nutrition and Clinical Services Division
NVD: Normal Vaginal Delivery
OR: Odds Ratio
ORS: Oral Rehydration Solution
WHO: Word Health Organization
